# Documentation of human rights abuses among Rohingya refugees from Myanmar

**DOI:** 10.1186/s13031-019-0226-9

**Published:** 2019-09-16

**Authors:** Rohini J. Haar, Karen Wang, Homer Venters, Satu Salonen, Rupa Patel, Tamaryn Nelson, Ranit Mishori, Parveen K. Parmar

**Affiliations:** 1 Division of Epidemiology and Biostatistics, University of California, Berkeley. School of Public Health, Berkeley, CA USA; 2 Yale School of Medicine, Section of General Internal Medicine, New Haven, CT USA; 3 New York University College of Global Public Health, New York, NY USA; 4 University of Massachusetts/Family Health Center of Worcester, Worchester, MA USA; 5 Department of Medicine, Washington University in St. Louis, St. Louis, MI USA; 6 Physicians for Human Rights, New York, NY USA; 7 Department of Family Medicine, Georgetown University School of Medicine, Washington, DC, USA; 8 University of Southern California, Los Angeles, CA USA

**Keywords:** Rohingya, Rakhine, Myanmar, Bangladesh, Genocide, Crimes against humanity, Istanbul protocol, Mass atrocities, Medico-legal evaluations, Scars, Survivors, Physical evaluations, Refugees, Injuries

## Abstract

**Background:**

Decades of persecution culminated in a statewide campaign of organized, systematic, and violent eviction of the Rohingya people by the Myanmar government beginning in August 2017. These attacks included the burning of homes and farms, beatings, shootings, sexual violence, summary executions, burying the dead in mass graves, and other atrocities. The Myanmar government has denied any responsibility. To document evidence of reported atrocities and identify patterns, we interviewed survivors, documented physical injuries, and assessed for consistency in their reports.

**Methods:**

We use purposive and snowball sampling to identify survivors residing in refugee camps in Bangladesh. Interviews and examinations were conducted by trained investigators with the assistance of interpreters based on the Istanbul Protocol – the international standard to investigate and document instances of torture and other cruel, inhuman, and degrading treatment. The goal was to assess whether the clinical findings corroborate survivors’ narratives and to identify emblematic patterns.

**Results:**

During four separate field visits between December 2017 and July 2018, we interviewed and where relevant, conducted physical examinations on a total of 114 refugees. The participants came from 36 villages in Northern Rakhine state; 36 (32%) were female, 26 (23%) were children. Testimonies described several patterns in the violence prior to their flight, including the organization of the attacks, the involvement of non-Rohingya civilians, the targeted and purposeful destruction of homes and eviction of Rohingya residents, and the denial of medical care. Physical findings included injuries from gunshots, blunt trauma, penetrating trauma such as slashings and mutilations, burns, and explosives and from sexual and gender-based violence.

**Conclusions:**

While each survivor’s experience was unique, similarities in the types and organization of attacks support allegations of a systematic, widespread, and premeditated campaign of forced displacement and violence. Physical findings were consistent with survivors’ narratives of violence and brutality. These findings warrant accountability for the Myanmar military per the Rome Statute of the International Criminal Court (ICC), which has jurisdiction to try individuals for serious international crimes, including crimes against humanity and genocide. Legal accountability for these crimes should be pursued along with medical and psychological care and rehabilitation to address the ongoing effects of violence, discrimination, and displacement.

**Electronic supplementary material:**

The online version of this article (10.1186/s13031-019-0226-9) contains supplementary material, which is available to authorized users.

## Background

Myanmar’s Rohingya people have been victims of decades of state-sponsored ethnic discrimination, detention, violence, and repression [1–7]. Although the Rohingya have lived in Myanmar for centuries, the country’s military junta enacted laws which restricted travel, limited educational and employment opportunities and barred access to citizenship for Rohingya people over the past four decades [4, 7, 8]. Rohingya people have also been subjected to forced labor, illegal detention, confiscation of land, and eviction, among other abuses [9, 10]. These restrictions have resulted, over time, in severely limited access to essential health and social services and a lack of political participation [5, 10]. Although Myanmar governmental policies have been “gradual and multidimensional … over many decades and have fluctuated in intensity,” the overall impact has been the isolation and persecution of the Rohingya and the systematic violation of their human rights [11].

Several waves of violence against the Rohingya have broken out over the decades, in 1942, 1978, 1991–2, 1996, 2012, 2016 and, most recently, in August 2017 [1, 12]. On August 24, 2017, a group of armed Rohingya men, part of the insurgent Arakan Rohingya Salvation Army, reportedly launched a raid on police outposts in the region, killing 12 members of Myanmar security forces.^12^ The Myanmar military, who had already moved troops and equipment into the area, initiated a statewide campaign of violence, which targeted unarmed Rohingya civilians living in Maungdaw, Buthidaung, and Rathedaung townships [12–14]. From August 25, 2017 through early 2018, hundreds of thousands of Rohingya people fled into Bangladesh, as many as 10,000 arriving in a single day [15].

Refugees described a campaign of organized, systematic, and violent eviction, including the burning of their homes and farms, beatings, mass shootings, sexual violence, executions, dumping of bodies in mass graves, and other mass atrocities [13, 16]. Many Rohingya arrived with fresh gunshot and machete wounds, burns, landmine injuries, injuries from sexual violence, and complex orthopedic injuries sustained in their home villages and during their flight to Bangladesh [13, 17]. The Myanmar government has denied a role in these attacks, and has claimed that Rohingya left voluntarily, burning their own homes and fabricating abuses [18]. As of June 2019, 750,000 of the 1.1 million Rohingya thought to have lived in Myanmar prior to August 2017 have fled to the Cox’s Bazar region of Bangladesh. These individuals live in what is now the largest refugee settlement on earth [19].

Human rights groups and media outlets have used satellite mapping and witness interviews to document these attacks [20–25]. They, along with various governmental and UN bodies, have cited this information to substantiate charges of crimes against humanity or acts of genocide but largely lack physical evidence [12, 17, 25–27]. Investigators with Physicians for Human Rights (PHR), an international advocacy organization based in New York, interviewed and examined survivors of the attacks in order to obtain physical evidence that substantiates narrative accounts of human rights violations. This methodology provides a unique capacity to correlate survivor narratives to physical findings. The objectives were to: (1) describe and document the nature of injuries and other physical sequelae resulting from events in Rakhine state, Myanmar; (2) assess whether or not physical findings corroborated the narratives of survivors; and (3) identify any patterns in the testimony and medical evidence to assess allegations of a systematic, widespread, and premeditated campaign of violence against the Rohingya.

## Methods

### Study design

This study was conducted by a collaborative group of investigators, including personnel at Physicians for Human Rights and U.S. based physician-investigators. Team members conducted an initial field visit to develop the research methodology and explore feasibility. Based on the initial scoping, we developed a descriptive case-study design to explore the potential human rights violations (and the physical evidence supporting those allegations) that led to the massive flight of Rohingya from Myanmar.

Our research team utilized purposive sampling to focus the study on recruiting interviewees with physical sequelae from the violence and snowball sampling to seek out further potential interview subjects. We included interviewees without specific physical injuries or scars who were willing to describe their experiences and/or provided information on the incidents. We collaborated with local organizations, health facilities, and community informants to identify initial interviewees and utilized their networks and contacts to recruit additional participants. The research team obtained informed oral consent from each interview subject following a detailed explanation of the purpose of the investigation and the potential benefits and risks of participation. If they provided consent, survivors were interviewed and examined utilizing a semi-structured interview and physical examination based on the Istanbul Protocol guidelines and principles [28].

### Data collection

Interviews and examinations were conducted in multiple locations throughout Rohingya camps in Ukhia and Teknaf upazillas (subdistricts), in the Cox’s Bazar district of Bangladesh. Interviews took place inside the refugees’ homes or in private rooms within health clinics in the camps or adjacent areas. Interviews were conducted during four field visits (December 2017, February 2018, March 2018, and July 2018) by six physician-investigators in mixed gender teams. All physician-investigators were trained in the physical examination, assessment, and documentation of injuries in persons who allege torture and ill treatment based on the Istanbul Protocol and had conducted multiple evaluations prior to the study. Interviews were conducted with an interpreter able to speak the Rohingya language (of both Rohingya and Bangladeshi origin, depending on the availability of interpreters), and whenever possible interviewees were matched with interpreters and physician-investigators of their preferred gender.

Physician researchers asked questions on violence experienced and injuries sustained in villages in Rakhine state prior to and during flight, including details on the mechanism of injury, any medical care obtained, and sequelae of the injuries, such as disabilities. Detailed physical examinations of scars resulting from gunshot wounds, blunt or penetrating trauma, burns, sexual violence, and any other physical injuries were conducted. Physician-investigators used sketches and photographs to document survivors’ scars. Given privacy and resource constraints, genital examinations were not conducted. Physician-investigators also assessed signs and symptoms of psychiatric distress, including depression and post-traumatic stress disorder. However, given time, privacy, linguistic and other resource constraints, full psychiatric evaluations, or the use of assessment instruments, were not conducted. Data were logged in notepads, and later transferred to password-protected computers. Images of injuries and scars, as well as of any available diagnostic data (such as copies of medical records and X-rays) were also taken using secure cameras and were stored in password-protected files.

### Analysis

Guided by the Istanbul Protocol guidelines and the UN Convention against Torture, physician-investigators determined consistency between narratives provided by respondents and their physical examinations [28, 29]. All interviews were manually coded into a database, which recorded demographic data, incident descriptions, types of injuries suffered, any ensuing disabilities, descriptions of witnessed abuses, and perpetrator identities.

### Assessments of consistency

We determined consistency between survivor narratives and physical findings based on the principles and guidelines of the Istanbul Protocol. Investigators evaluated how each injury was sustained, characteristics of any resulting wound, such as depth, shape, scarring pattern, time frame of the healing pattern, and any additional scars that resulted from secondary infection or surgical treatment. We corroborated this information with the testimony of the individual and any additional data or medical records in order to make an assessment of consistency for each individual wound and scar and then for the evaluation as a whole.

### Ethical considerations

Prior to field research, exploratory qualitative work, including literature review, interviews with local health workers, assessment of medical documentation, and meetings with local and international stakeholders, was conducted to ensure that the research was necessary, feasible, and welcomed by the community.

Given the vulnerability of the respondents both to outside persecution and to possible re-traumatization, the research team gave high priority to the ethics of the research and the safety of the refugees. Privacy protocols ensured that adults were interviewed alone or in the company of those they specifically requested to participate, and children under age 18 were interviewed only in the company of their parent or guardian. All adults provided verbal consent prior to the interview and physical examinations. Minors under the age of 18 gave assent, while their parents and/or guardians gave consent.

Photographs and videos of the survivors required separate written consent. Individuals could refuse or stop participation without any negative consequences. If there were signs of distress or discomfort during the interview or examination, physician-investigators followed a protocol to seek continued consent to participate, take breaks during the interview and examination, and communicate with the respondent on their preferences. When deemed appropriate and/or necessary, the physician-investigators referred respondents to appropriate psychiatric and/or medical care. PHR’s Ethical Review Board (ERB) approved this research.

## Results

**Fig. 1:**
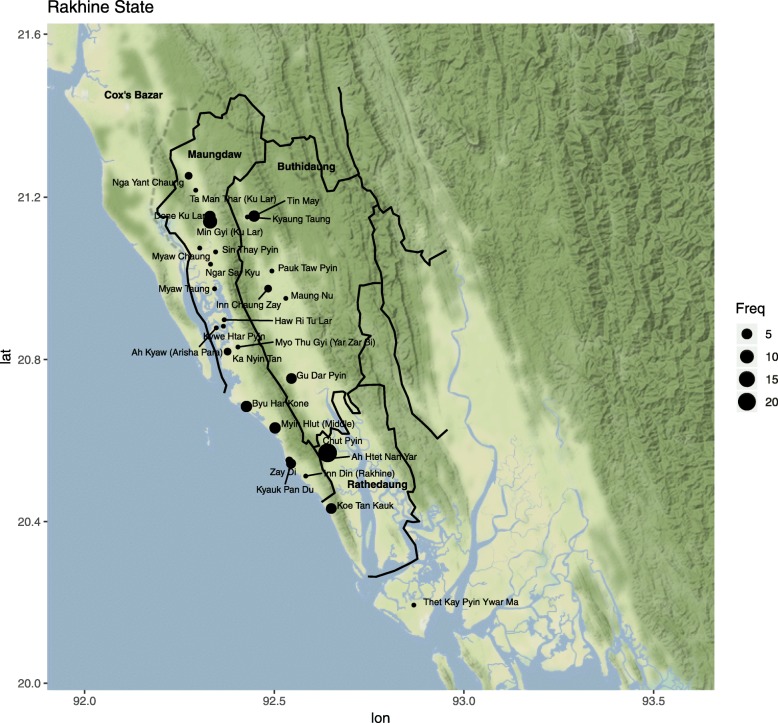
Village of Origin of Participants

A total of 120 survivors were asked to participate in the study between December 2017 and July 2018 on four separate field visits. Six people declined and 114 survivors were interviewed. A total of 78 men (68% of all participants) and 36 women (32%) were interviewed. Eighty-eight adults (range: 18–74 years old; 77% of all participants) and the parents of 26 children (range: six months - 17 years old; 23%) were interviewed, along with the children where age-appropriate. 90 (78.9%) of all participants had injuries as a direct result of the attacks in Myanmar. The other 24 (21.1%) participants provided descriptions of their experiences and/or what they witnessed (Additional file 1). Figure 1 illustrates reported place of origin and Table 1 provides demographic information.

**Table 1:** Demographic summary of participants

Age Group	0–10	11–18	19–59	60+	All
Number of People	11	15	80	8	114
Number of Males	7 (63.6%)	11 (73.3%)	56 (70%)	4 (50%)	78 (68.4%)
Number of Females	4 (36.4%)	4 (26.6%)	24 (30%)	4 (50%)	36 (31.6%)
Number of People Injured	11 (100%)	13 (86.7%)	61 (76.3%)	5 (62.5%)	90 (78.9%)
Number of People Missing Family Members	6 (54.4%)	7 (46.7%)	32 (40%)	3 (37.5%)	48 (42.1%)

The percentages in parentheses denote the percentage of people in the corresponding age group who fall into the corresponding category. The categories are not mutually exclusive

The following analysis reports on three facets of our data gathering: (1) themes among the narrative interviews; (2) patterns among the physical examination findings; and (3) assessments of consistency.

### Narrative interviews

When asked to describe events leading to their flight to Bangladesh, respondents related testimonies of massacres of villagers, burning of homes, sexual violence, and attacks on children. They described an organized and systematic expulsion from their villages that was consistent within and across villages in northern Rakhine state. Several facets of the attacks emerged as consistent themes from the survivor interviews, including patterns in:

1. the persecution, arrest, and detention of Rohingya residents prior to the main attack;
2. the organization and execution of the attacks;
3. the participation of non-Rohingya civilian vigilante groups;
4. the purposeful destruction of homes, villages, and livelihoods and ensuing eviction of the Rohingya residents;
5. the delayed, denied, and/or inadequate medical treatment of injuries in Myanmar; and,
6. the events during flight from Myanmar to Bangladesh.

We described the similarities and patterns of attack under these themes and included specific quotations and examples.

#### Persecution, arrest, and detention

Several testimonies discussed arrests of Rohingya individuals before and during the wave of attacks. Two of the interviews were conducted with men who reported being taken from their villages by soldiers before August 26 and detained and tortured as part of ongoing military actions against the Rohingya. These men were part of a group of 67 men reportedly taken from their villages in mid-August, detained, and tortured during interrogations about political or terrorist affiliations until they were able to pay for their own release. Arrest was also mentioned in other encounters as part of routine persecution of Rohingya, justified as “anti-terrorism” clearance operations by the Myanmar authorities.

*“[When I was imprisoned], I saw one of the men killed during this time when a soldier hit him with a large timber in the head. Several days later, I passed a place where I could see into a room where the body of this man was laying on a table and had been cut open to the heart. I was told that I would need to pay 400,000 [Myanmar kyats (about* £*217 GBP)] for my release and when I promised to go home and return with the money, I was released after seven days. Soldiers said that they meant for all of the men to die and that only payment of money would keep them alive.”* **[Male, 67]**

*“One day, months before August, the military came to my village and said “you are terrorists” and took several villagers to jail. The military came and took pots and several household items from us. They were very rough with us and would not let us pray.”* **[Female, 60]**

#### Organization and execution of the attacks

Respondents described similar patterns in how the perpetrators entered and surrounded a village. In some cases, loudspeakers ordered the Rohingya to exit their homes and present themselves. Soon afterwards, the military and civilian militia members started shooting into the homes and raiding them, both from inside vehicles and on foot, to drive out any remaining dwellers. Homes were looted for any valuables and then set on fire, destroying entire villages. Many survivors recalled the chaos of those events, when people became separated from their families and were beaten or shot while trying to escape into nearby forests or fields. In many cases, remaining villagers were then gathered into a central area where men and women were forcibly separated. Men were beaten and executed, and women were taken into specific central buildings like schools, where they were raped, mutilated, and/or killed. Attacks lasted several hours, with the attackers sometimes going as far as checking surrounding fields for any survivors before leaving the area.

Participants whose villages were attacked in the afternoon reported that they saw soldiers beginning attacks on neighboring villages in the morning before making their way to adjacent villages. In multiple narratives, respondents recalled that helicopters, troops, and other materials were moved in advance of the attacks, suggesting advanced planning of the alleged attacks on August 24.

One participant reported: *“They came early in the morning. The day before, they were in the neighboring villages at the same time and we could see the smoke [from there].”* **[Male, 20]**.

Another stated: “*From this hill where we are now, we see many villages being burned and smoke rising over our country.”* **[Male, 19]**

One participant described fleeing from one village under attack to another that was also soon attacked: *“After we fled to the jungle, we returned and found burning homes and dead bodies. From [my village], we fled to [a neighboring village]. Here the military tried to corner us and bomb us with helicopters. But we fled again. We went to [one village], then [another], and ultimately [another village] near the border. There was no food and no rest on the way to the camps.”* **[Male, 20]**

#### Participation of non-Rohingya civilian vigilante groups

Interviewees frequently described that non-Rohingya civilians participated in the atrocities in concert with the Myanmar military. The civilian groups were described by the participants as “Buddhists” or “monks” or as “Buddhist villagers.” Differing from soldiers, civilians were identified by a variety of characteristics, including lay clothes and lack of uniforms. They used farming equipment, such as machetes, during the attacks. Many respondents reported that they recognized civilian attackers as neighbors. Rohingya respondents described military entering villages alongside groups of civilians. As the military surrounded the village, made announcements for Rohingya residents to present themselves, and fired on villagers and their homes, the civilian vigilante groups were permitted to loot the homes of Rohingya and set them on fire. They also often were seen cutting men’s throats with their machetes and other sharp farming tools.

*“When I came out of my house, I saw Buddhists setting houses on fire on one side of the village and soldiers shooting at the houses and at people.”* **[Male, 28]**

*“The attack initially was by uniformed military and Buddhists. The military were wearing greenish/khaki uniforms. Fifty soldiers and 100 Buddhists arrived at the village and then split up and killed us by shooting and stabbing.”* **[Female, 40**]

#### Destruction of homes and livelihoods and eviction of the residents

There were numerous testimonies of military and vigilante groups firing bullets directly into homes or setting them on fire while people were still inside, resulting in death and injury. Many refugees reported living in wooden homes that were highly vulnerable to arson and easily penetrable by bullets. Homes were also looted by non-Rohingya civilians, who stole valuables such as jewelry. Mosques were often destroyed, as well as crops and other forms of livelihood.

*“They [the non-Rohingya civilians] took everything metal out of my house – the pots, the spoons, the jewelry. Then they burned it down.”* **[Female, 15]**

“*I saw soldiers burning homes that had people inside them and I heard screams from many women and children who died in their homes while on fire.”* **[Male, 18]**

***“****I saw military soldiers and Buddhist civilians enter my village. Once they entered the village, I saw the military start shooting at houses with their rifles. I stayed in my house and heard houses being set on fire and shooting from outside. I was able to look out the window and see soldiers and Buddhist civilians taking the goats and cows as well as shooting into houses. After an hour, I and my family decided to run from [our] house and, as we did, we were stopped [and attacked]. … Both Buddhist civilians and soldiers yelled at me and other Rohingya: “Leave and never return, this is not your home” and “You are Bangladeshi, this is our country, not your country.”* ”**[Male, 50]**

#### Delayed, denied, and/or inadequate medical treatment of injuries in Myanmar

Several refugees noted that both Muslim and Buddhist health care providers refused care to Rohingya during and after the August 2017 attacks for fear of being persecuted or targeted themselves. Some respondents described attempting to reach local health workers to treat their wounds but being turned away. As a consequence of the denial and delays in medical treatment, many respondents noted that their injuries worsened and healed poorly. Based on our physician-investigator assessments, in many cases these delays resulted in permanent disabilities. We describe these physical examination findings in the following section.

*“I went to three doctors and each of them said, ‘No, get out of here.’ The last one gave him [my son] a little medicine but told me never to come back.”* **[Male, 55]**

A man who had chronic health conditions said: *“They can’t help us. If they do, people will think they are Muslim and kill them …*. *Even our own Rohingya doctors won’t help us. They are so afraid. But then, what hope do we have of going back?”* **[Male, 23]**

#### Events in flight

In addition to violence within the villages, respondents described targeted violence during their flight to Bangladesh. To cross the border from their home villages, many survivors noted they had to travel several days on foot over difficult terrain, through jungles, over mountains, and across rivers. Respondents described that the roads were patrolled by Myanmar security forces and often littered with landmines, rendering the area unpassable. Even while traveling through the jungle, several respondents noted that they were shot at by the military when they were seen. Respondents also reported being injured during their flight and developed wound infections, new lacerations, or other injuries from being trampled by wild animals. They also reported deterioration of chronic conditions.

*“We and other villagers began to run from the hamlet and could see the Myanmar military from a distance. As we crossed out of the hamlet, some villagers stepped on landmines. Three people near me stepped on mines and died immediately.*” **[Male, 30]**

“*The military were trying to surround the village and burning homes. I awoke my family and ran into the forest with them with nothing but my lungi and shirt. An hour later, the army started firing randomly into the forest, knowing residents were hiding nearby.”* **[Male, 28]**

*“We heard the military were going to kill us soon, so we left our village. Just before we arrived at border, we were caught trying to leave by military. I was beaten on the leg with a large gun. My nephew was beaten on the head and stomped on the neck and died there. The others were able to run away and get to the border.”* **[Female, 25]**

### Physical examination findings

Among the 114 respondents who were interviewed, 90 (78.9%) reported sustaining physical injuries as a direct result of violent attacks at the hands of Myanmar security forces. This high proportion of injuries is an expected result of our purposive sampling methodology seeking those with injuries. The types of physical injuries sustained and mechanisms described revealed the following common patterns:

1. injuries from projectiles, such as gunshot wounds;
2. blunt force trauma, such as beatings;
3. penetrating trauma from knives, machetes, and other sharp objects;
4. injuries secondary to explosions, such as blast trauma and hearing loss;
5. burn injuries from fire;
6. injuries from sexual violence;
7. psychological sequalae of surviving violent traumatic incidents.

We found that, in all cases, there was a high level of consistency between the physical examination of the injuries and the description of how they were sustained and the weapons employed. That said, in one case, an interviewee described a number of injuries that were intentionally inflicted, several of which were highly consistent with the mechanism of injury but two of which were consistent with non-traumatic stretch marks. Of note, it is common in forensic evaluations for survivors to forget the origins of some scars that investigators identify, or for other scars to be attributed to an injury when they are not related, without ill intentions. Other than this one case, all of the survivors we interviewed had physical findings that were entirely consistent with their narratives. We describe patterns among the narrative descriptions of events leading to physical injuries among all respondents and injury patterns among those we evaluated. Examples of consistency assessments (with identifying data removed) are given with the relevant case narratives. Summary data is presented in Table 2.

**Table 2:** Injuries, disabilities, and symptoms among respondents

Age Group	0–10		11–18	19–59	60+ All	
Number of People	11		15	80	8	114
Gun Shot Wound	8 (72.7%)		9 (60%)	44 (55%)	2 (25%)	63 (68.4%)
Blunt Force Trauma	1 (9.1%)		3 (20%)	19 (23.8%)	2 (25%)	25 (21.9%)
Injury from Fire or Explosion	5 (45.5%)		6 (40%)	29 (36.3%)	2 (25%)	46 (40.4%)
Sexual Assault	0 (0%)		0 (0%)	4 (5%)	0 (0%)	4 (3.5%)
Permanent Disabilities	6 (54.5%)		6 (40%)	35 (43.8%)	0 (0%)	47 (41.2%)
Symptoms of Psychological Trauma	0 (0%)		2 (13.3%)	20 (25%)	1 (12.5%)	23 (20.2%)

The percentages in parentheses denote the percentage of people in the corresponding age group who fall into the corresponding category. The categories are not mutually exclusive

#### Injuries from projectiles (gunshot wounds)

Physical findings consistent with gunshot wounds were present in 63 survivors. These injuries were reportedly from rifles, shotguns, and other projectile weaponry. The ages of persons with these examination findings ranged from 2 years old to 60 (17 were children). These narratives almost uniformly describe being shot while attempting to flee attacks, and the location of these wounds often revealed entrance wounds to the posterior body parts (the back, back of legs, etc.) of the individuals. The age and appearance of the wounds on all survivors were consistent with the descriptions they gave and the timeline of the incidents in Myanmar. Survivors described being shot while running from their villages, as well as when they were hiding in nearby forests and fields:

*“My house was surrounded by soldiers with rifles, so we started running away from the village. As I was running, I was shot in my lower leg from behind and fell, but my family helped me and carried me away.”* **[Male, 45] The survivor’s injuries and the presence of bullet fragments in his leg as well as his limited movement in the affected leg were consistent with his report of being shot with a rifle while fleeing.**

*“I was sleeping when I heard gun shots. I went out to the road and saw security forces about a half kilometer away firing weapons. On the other side of the road, security forces were trying to surround the village and were burning houses. I awakened my wife and children and ran into the forest with them. I had nothing but my longyi (cloth wrap) and shirt. As we were hiding, security forces started firing randomly into the forest, knowing that residents were hiding nearby. My nephew was shot in the chest and died immediately. My cousin was shot in the back of the head and died immediately. I was shot and I fell. I was losing blood and feeling weak.”* ***[Male, 28] The survivor’s external scars and persistent weakness and limited range of motion were consistent with his description of his trauma.***

*“When I was praying in the mosque, I heard gunshots. I ran back to my house but as I was running back, I was shot both twice. My father and brother were with me and they helped carry me to beside the river. While [we were] in [the] forest, my father returned to the house and brought back all the others. After about four days, we tried returning to the village to see what was happening; I saw houses burning, saw the Rohingya people running around, and felt very afraid. We walked 10 days and arrived at the hospital in Bangladesh, where I stayed for four months and had surgery.”* ***[Male, 19]. He has significant atrophy to the limb that was injured, with limited range of motion and weakness. There are visible gunshot entry and exit wounds as well as a surgical wound on that limb. The other gunshot wound has an entry wound with a nearby surgical scar that are consistent with his description of sustaining two gunshot wounds requiring surgery.***

*“The military surrounded the village and Buddhists were entering all the homes and looting all the valuables. If people resisted or ran away, the military attacked them. After this, they started burning the homes in the village. I ran into the forest with my family and was shot in the [limb]. I tried running a bit further and then I think I fainted. When I woke up, no one was around and [the] village was destroyed. I have not seen [my] parents since then. I found an uncle also in the forest and two siblings and they lived in the forest two months, subsisting on “forest leaves.” We were too afraid to leave, but, eventually, my uncle found a way to get to border. I had no treatment for [my] arm and was not able to move it and had severe pain. At the border, Bangladeshi officials referred me straight to the hospital, where they amputated my [limb].*” ***[Male, 15]. He has a total amputation of the limb with a crusted eschar that is not completely healed yet, consistent with his description of his injuries, lack of timely treatment, and subsequent infection requiring amputation.***

#### Blunt force trauma

Physical findings consistent with intentional blunt force trauma were present in seven cases. The reported causes of blunt force trauma included being struck with rifle butts, batons, sticks, or rocks, being kicked and whipped with wire, or tied with ligatures that caused trauma.

These experiences occurred in both children and adults. One survivor, a five-year-old girl, sustained permanent pelvic and spinal damage after being thrown against a stone wall and has not been able to walk since then. A man reported that he and other men were beaten with rifle butts by soldiers; he had scars consistent with his description. Other respondents described blunt force injuries:

*“My hands were bound behind my back. Four or five soldiers took turns striking me with their rifle butts. I was hit many times. I begged them not to kill me and promised I would go to Bangladesh and never come back.” This respondent said they began to beat other men in the same manner and that they all pleaded not to be killed and promised to leave Myanmar and not return.* **[Male, 35] His physical exam findings, with skin wounds and persistent swelling and deformity around multiple displaced fractures of the leg, along with X-rays that were reviewed, were consistent with his account of being severely beaten with rifle butts.**

*“I was beaten on the leg with a rifle butt as we were leaving our village. They starved us and then tried to kill us. Pus was coming out of my leg by the time I got to the hospital [in Bangladesh].”* **[Female, 25]. She has a large healing large wound with a surrounding area that appears scarred from a previous infection and trauma consistent with her history of inadequate early treatment of a significant blunt trauma.**

#### Penetrating trauma

Six survivors reported being attacked with knives, machetes, and other sharp objects during the attacks and all had physical findings consistent with their testimony. Five respondents had injuries from stabbing and other knife injuries. One had injuries secondary to an attempt to cut his/her throat.

*“What happened is always part of my mind. The soldiers kicked me with their legs, beat me with a flashlight on my back and shoulders, and used a short knife to stab me. I felt the knife drag along my skin. I tried to grab it and it cut my finger as well....”* ***[Female, 30] She has permanent disabilities to the hand and knife wound scars that are consistent with the violence she described.***

*“I saw my children stabbed by a long knife and then I was knocked out. When I woke up I was in a room in a small house with dead women and children all around and everyone was stabbed. The house was on fire. I ran out of the house and fled to a forest area nearby. I was bleeding from [my] neck area.”* ***[Female, 25] She had two linear scars that were consistent with knife wound injuries as she described.***

#### Blast injuries

Thirty-seven of the survivors described the use of bombs and other explosive devices. Fourteen survivors described personal injuries from these blasts, and all had physical findings consistent with their testimony. Various terms were used to describe devices thrown by hand, such as grenades, or launched projectiles such as rocket-propelled grenades, commonly called “launchers.” Injuries included primary (hearing and vision loss), secondary (neurovascular injuries from flying debris), tertiary (broken bones from being thrown), and quaternary injuries (burns after a house was bombed). As is typical with blast injuries, survivors frequently had injuries to multiple body systems.

*“I was in the kitchen of my home when the attacks started. Eleven other family members were also in the house at that time. As I began hearing shouting outside, I was hit by something hot and heard a loud explosion at the top of my house. I fell to the ground and was confused. The next thing I remember is being picked up and helped by family members to run out of the house and away from the village. I was in severe pain and my house was full of smoke and flames. When I got outside, I could not see well because of the smoke and flames from other homes.”* **[Male, 20] The physical examination reveals numerous injures that are consistent with his account of blast injuries secondary to an explosive/incendiary device being detonated in or on his home, including permanent vision loss and disability.**

*“When I heard the gunfire, I came out of [my] house and tried to flee. I realized my eight-month-old son had been left in the house. I went back to get him and saw that the military were shooting rocket grenade launchers at the fleeing people and setting houses on fire. As I ran back out of [the] house, fire hit me in my chest. Something from the rocket launcher hit me in my face and head. My face, hair, lips, ears, and beard were all burned. My son and I fell to the ground. I tried to get up and fell again. I lost consciousness. After about an hour, the military went a short distance away from the village and my family found us in a paddy field and took us away*.” **[Male, 25] His injuries were highly consistent with an explosion, with blast, burn, and shrapnel injuries as described. The extensive scarring makes it difficult for him to work or carry out his daily activities.**

#### Burn wounds

Physical findings consistent with burn injuries were present in three cases. Burns were sustained when homes were set alight or as a result of an attack with an explosive device. Burn wounds were notable for extensive scarring and disability, as well as chronic pain.

*“It was a Sunday at 10 a.m. and the I was just finishing putting away the breakfast. My [adult] daughter was outside fetching water from a nearby pump. The [Buddhist villagers] set fire to the house as the soldiers were standing outside. I was trying to run out of the burning home and tripped and fell and embers fell on me before I could get up and run out. My daughter ran deeper into the fields. She came back for me and poured water on my burns later when the military went to another house in the village.”* ***[Female, 60]. She has recently-healing burn wounds that appeared deep and extensive with resulting scarring and difficulty with range of motion consistent with her description of not having been able to leave the burning area quickly enough.***

In a lengthy description of events, one girl described:

*“They took me and my mother and sisters to a hut …*. *They bashed me on the head and I passed out … I woke up to a strong smell of burning and pain all over my body. I realized that my hands, feet, and back were in flames and I crawled out of the house and made my way to the cover of nearby trees … My sisters and my mother died in that burning hut … I lost my whole family.”* ***[Female, 15]. The examined portions of her skin revealed extensive scarring from burns, with hypertrophic areas of varying depth. The physical locations of the scars are consistent with her description of how she was lying and of waking up to feeling burned on the exposed areas. The injury on her head is consistent with the mechanism she described.***

#### Sequelae of sexual violence

Sexual assaults, breast mutilation, and rape by both Myanmar military and non-Rohingya civilians were frequently described, both before and during the August 2017 attacks. Three women descripted being raped and two described suffering other types of sexual violence. Urogenital exams were not conducted as part of this study. The descriptions of sexual violence often include multiple women being gathered into specific areas (a hut, a school, etc.) and soldiers and non-Rohingya civilians participating in the assaults.

*“I was taken in a group of six to seven women by the military into a home. When I entered the home, I was hit on the head with a stick and knocked to the ground, but I remained conscious. My three-month-old baby was in my arms, and the military sliced my child with a machete and killed her. After I was hit on the head, I was raped. There were six women and six military men who raped the women. The women fought against this and were beaten severely. After they were beaten badly, they were undressed and raped. This is when [my limb] was broken. Each [woman was] raped by one man. After I was raped, the man who raped me used a knife to cut [me] in two places. I was left for dead, and many of the women around me were dead.”* ***[Female, 40]. Her bony deformity and laceration scars are consistent with her description of events.***

One survivor recounted that she was taken to the local school, where many other young women were being held.

*“We were all told to lie face down. When I tried to resist, I was thrown to the ground and beaten. Before letting me go, one soldier stabbed me with a knife at the tip of his rifle. Many women around me were being raped at the same time by both soldiers and civilians. I was hit on the head, and, when I awoke, many of the women around me had been mutilated and killed. I crawled away when they were not looking.”* ***[Female, 20]. She sustained a laceration that is consistent with her description of the incident that is now healed but has scarred permanently.***

In several instances, respondents described sexual violence, including seeing women with breast mutilation, rapes, and the targeting of women for other violence:

*“Her husband chased after the soldiers and was shot dead. [One woman] was raped by multiple soldiers and died of her injuries. My whole family was terrified. Who was going to be next?”* **[Female, 20]**

*“The military had come into the village at random and taken girls and women, some as young as eight. They took them out into the field. Many soldiers would rape the girl. Some were mutilated, beheaded, or otherwise abused. None of the women or girls ever came back. It occurred at random. One month before the incidents above, a neighbor was alone in her home with her baby and the husband was in the fields. The military came and raped and murdered her. The baby was trying to nurse the dead mother’s breast and they beheaded the baby.”* **[Female, 60]**

One woman described that:

*“The military came and took my 35-year-old pregnant cousin and her husband from the village into the rice fields. They killed the husband immediately and raped my cousin. They cut off her breasts and then killed her and left her in the fields.”* **[Female, 20]**

#### Psychological sequalae

Many of the narratives above and throughout the interviews highlight the fear, distress, and terror that the attacks caused. While investigators were unable, due to resource and time constraints, to make definitive diagnoses of psychiatric disorders, they noted that nearly all survivors demonstrated psychological impacts of violence experienced. Some survivors described persistent post-traumatic symptoms such as insomnia, intrusive thoughts, strained relationships, depression, and anxiety.

One respondent noted that she cannot sleep and is constantly thinking of what happened:

*“My heart is burning for my children, for my husband. They made me alone …*. *I had my husband and six children, two boys and four girls. All were killed. I keep thinking that my baby was still alive in the house when they burned it down.”* ***[Female, 40]***

Another respondent was tearful throughout the interview and alternated between reporting what happened, when asked, and staring blankly for extended periods of time:

*“I will never forget what happened to me. I am always thinking about it …*. *The military surrounded my house and ordered me to come out. I was carrying my baby and running with my two- year-old son. They were shooting at us and a bullet killed my baby and then entered my [body].”* ***[Female, 20]***

## Discussion

This study is unique in going beyond survivor narratives to identify physical evidence of violence and abuse to corroborate possible human rights violations. We systematically document the physical findings of 114 Rohingya refugees interviewed and examined in refugee camps in Bangladesh in the months following the massacres that began in August 2017. Overall, the patterns, consistency, and multiple independent confirmations of the individual testimonies reflect a consistent picture of intentional, brutal violence suffered at the hands of soldiers, often in concert with non-Rohingya civilians. Injuries included those from gunshots, beatings, knives, and other sharp instruments, explosions, fire, sexual and gender-based violence, blunt trauma, and other types of trauma related to the violence. We documented 63 cases of civilians surviving gunshot wounds, suggesting that only those with large-scale access to these weapons, namely, state actors such as the military or police, were likely to have perpetrated these attacks. The consistency between these narratives and the injuries, scars, symptoms, and disabilities documented by the investigators represent unique physical evidence of human rights abuses among Rohingya refugees from Myanmar. The findings illustrate the range of atrocities that were committed and support allegations of human rights violations that would fall under the Rome Statute of the International Criminal Court’s (ICC) definition of crimes against humanity. In particular, these findings are consistent with the assessment by the UN Fact-Finding Mission (FFM) that the attacks were “widespread or systematic” as stipulated by the definition of crimes against humanity and conducted “with intent to destroy, in whole or in part, a national, ethnical, racial or religious group” as established by the ICC definition of genocide [25, 30].

While each survivor’s experience was unique, similarities in the experience of Rohingya citizens prior to the attacks, the organization and execution of the attacks, the involvement of non-Rohingya civilians, and the purposeful destruction of homes and villages support allegations of a systematic, widespread, and premeditated campaign of forced displacement and massacre. The numerous reports that describe similar experiences of how the Myanmar military raided villages, burned homes, and killed and expelled the residents illustrate the systematic nature of these assaults. Demographic characteristics of the survivors support the allegation that these crimes were widespread. Survivors, including men, women, children, and the elderly, came from 36 different villages within Rakhine state, and were dispersed geographically over the state, strongly supporting that these attacks targeted a civilian population. The forensic evidence, consisting of physical examinations of the survivors paired with the corresponding narrative, indicates that since August 2017, Rohingya men, women, and children from Myanmar suffered physical and sexual brutality.

This study describes a human rights-based process to corroborate physical evidence with multiple narrative accounts, following the Istanbul Protocol methodology. It highlights the presence of physical evidence that has thus far not been described or documented through other means, given the Myanmar government’s lack of cooperation with independent investigations such as the search for and exhumation of mass graves. These physical data can augment and corroborate forensic architectural analysis and open-source investigations that have utilized satellite data and social media.

### Limitations

There are several important limitations to this work. The sample size and purposive sampling methodology was intended to explore the range of injuries, establishing the geographic, temporal, and legal scope and scale of the abuse, and to gather physical evidence of reported human rights violations. This study was not designed to provide prevalence of specific types of injuries, or of exposure to violence. To our knowledge, there are no reliable estimates of how many people have been injured during these massacres. Related epidemiological research by PHR and other groups has established conservative estimates that between 7800 to 9400 people died as a result of the August 2017 violence and subsequent displacement [31, 32, 43].

There were also a number of practical constraints that may have affected our findings. None of our physician-investigators spoke the local language. It is possible that we missed details of the narratives and testimonies or that the survivors were less forthcoming when necessity required the use of Bangladeshi interpreters or investigators of the different gender. The research was conducted over four separate field visits over 8 months, with possible differences in the survivors’ recall of events, and in the appearance of scars and other injuries suffered by those interviewed earlier and later. We note that in our analysis, the dates of the injuries were taken into account in assessing the scarring pattern. Documenting genital physical evidence of sexual violence and the psychological consequences of these traumas was beyond the scope of this investigation, given privacy concerns, resource constraints, and concerns over re-traumatization. Although we ensured that all interviews took place in a private space, the thin walls in refugees’ homes in the camps and medical clinics, often made of plastic sheeting, did not always provide complete auditory privacy. This factor may have also affected the survivors’ willingness to share their narratives, particularly those of sexual violence.

This study had a smaller group of women respondents, possibly due to cultural constraints and limitations in our sampling methodology. Further, the conservative culture and status of women may have affected the willingness of women to speak with outside investigators, and snowball sampling may have led to introductions to more men than women. This factor may have contributed to an underrepresentation of gender-specific violence, such as rape.

### Challenges ahead

Critical data on the scope and organization of the violence, the breadth of injuries, and the manner in which civilian and military forces coordinated, utilized weapons, destroyed villages, and evicted hundreds of thousands of Rohingya citizens have now been established by this manuscript and other recent publications [31, 32, 43]. Additional work is needed to assess the incidence of specific acts and issues, such as sexual violence and its sequelae and ongoing mental health concerns. The multiple reports of disabilities in this study resulting from the attacks mean that these individuals require ongoing care and rehabilitation services – resources that have been difficult to access in the setting of a refugee camp and in a low-resource country such as Bangladesh.\The scarcity of rehabilitative services as well the lack of inclusive programs, in tandem with a difficult and inaccessible geographical terrain and stigmatization of those with physical, mental and psychological disabilities, has resulted in fundamental barriers to disabled persons exercising their basic human rights and participating fully in their community [33–38].

Nearly 1 million Rohingya people now live in Bangladesh with insufficient aid, poor infrastructure, and no durable resolutions or protections in sight. There is a critical need for advocacy, policies, and services for these communities that have already suffered unthinkable trauma and live in perilous conditions. Moreover, an estimated 600,000 Rohingya still live in Myanmar, with more than 100,000 of these living in internment camps [19, 39, 40].^25^ Human rights for the Rohingya population have been severely restricted in Myanmar for decades and this situation is likely worsening as Myanmar authorities continue to deny international observers and aid groups access to these areas. Within this context, health workers and researchers should continue advocating for the Rohingya, drawing attention to their plight and ensuring that humanitarian assistance such as food, nutrition, clean water, education, and health care is provided, consistent with fundamental principles of human rights.

This study’s findings, documentation, and conclusions are a valuable contribution to calls for the UN Security Council to refer Myanmar to the International Criminal Court and other credible accountability mechanisms for crimes against humanity and genocide. While Myanmar is not a state party to the ICC, it ratified the Convention on the Prevention and Punishment of the Crime of Genocide (commonly referred to as the Genocide Convention) in 1956 [41]. The ICC confirmed that it may exercise jurisdiction over these incidents because an element occurred in Bangladesh (which has been an ICC member since 2010). Accountability processes may include: (1) urging all UN member states to provide political and financial support to the international accountability mechanism created by the UN Human Rights Council in September 2018 through resolution A/HRC/39/L.22; (2) demanding unfettered access to Rakhine state for independent monitors, international human rights organizations, journalists, aid agencies, and other international observers; (3) ensuring that any Rohingya repatriation is not implemented without actionable guarantees and sustainable conditions for safe, dignified, and voluntary return for the Rohingya; and (4) imposing bilateral and multilateral sanctions, including arms embargoes against the Myanmar military and targeted sanctions against individuals responsible for crimes and serious abuses [42]. Recent decisions by the ICC on jurisdiction, the U.S. House of Representatives condemning the genocide in Myanmar, and the establishment of independent investigations of the situation by the UN Human Rights Council (including the Independent Investigative Mechanism for Myanmar and the Independent International Fact-Finding Mission on Myanmar) are promising and have given momentum to the quest for justice and to ensure that the Rohingya are not forgotten [25, 42].

## Conclusion

This study provides evidence in support of allegations of systematic and widespread attacks on the Rohingya in Myanmar. Based on detailed narrative interviews and corroborating physical evidence gleaned from the wounds and disabilities of survivors, we conclude that there have been a range of violent abuses against the Rohingya people. Documentation of these abuses supports calls for the UN Security Council to refer Myanmar’s military leadership to the International Criminal Court or other credible accountability mechanisms for crimes against humanity and acts of genocide. This study has substantiated the need for these measures and calls crucial attention to the strength and endurance of the Rohingya people in the face of persecution.

## Additional file

Additional file 1: **Table S3.** Information on Crimes Witnessed. (DOCX 22 kb)

## Data Availability

The datasets generated and/or analyzed during the current study are not publicly available to protect the security of the survivors and their families. Summary data are available from the corresponding author.

## Abbreviations

ICC: International Criminal Court
Istanbul Protocol: The Manual on Effective Investigation and Documentation of Torture and Other Cruel, Inhuman or Degrading Treatment or Punishment, commonly known as the Istanbul Protocol
PHR: Physicians for Human Rights
Rome Statute: Rome Statute of the International Criminal Court
UN: United Nations
