# Supplementary material for: Documentation of human rights abuses among Rohingya refugees from Myanmar
Source: Confl Health. 2019 Sep 16;13:42. doi: 10.1186/s13031-019-0226-9 (PMC6745767; doi:10.1186/s13031-019-0226-9)
Supplement: Supplementary file 1 — Table S3. Information on Crimes Witnessed. (DOCX 22 kb) [file 13031_2019_226_MOESM1_ESM.docx]

**Additional file 1: Table S3**

**Information on Crimes Witnessed**

| *Age Group* | *0-10* | | | *11-18* | *19-59* | | *60+* | | *All* |
| --- | --- | --- | --- | --- | --- | --- | --- | --- | --- |
| *Number of People* | | 11 | 15 | | 80 | 8 | | 114 | |
| *Witnessed Murders* | | 6 (54.5%) | 12 (80%) | | 53 (66.3%) | 7 (87.5%) | | 78 (68.4%) | |
| *Saw Corpses* | | 6 (54.5%) | 13 (86.7%) | | 57 (71.3%) | 7 (87.5%) | | 83 (72.8%) | |
| *Witnessed Shooting** | | 6 (54.5%) | 11 (73.3%) | | 67 (83.8%) | 6 (75%) | | 90 (78.9%) | |
| *Witnessed Arson* | | 5 (45.5%) | 9 (60%) | | 56 (70%) | 3 (37.5%) | | 73 (64%) | |
| *Witnessed Theft* | | 0 (0%) | 6 (40%) | | 20 (25%) | 2 (25%) | | 28 (24.6%) | |
| *Witnessed Sexual Violence* | | 0 (0%) | 1 (6.7%) | | 16 (20%) | 5 (62.5%) | | 22 (19.3%) | |
| *Witnessed Any Violence or Crimes* | | 7 (63.6%) | 15 (100%) | | 75 (93.8%) | 8 (100%) | | 105 (92.1%) | |

NOTE: The percentages in parentheses denote the percentage of people in the corresponding age group who fall into the corresponding category. The categories are not mutually exclusive.

* “Witnessed Shootings” indicates that a respondent saw attackers firing guns in a targeted or indiscriminate manner. It does not necessarily indicate murder.
